# Health insurance for patients with HIV/AIDS in Vietnam: coverage and barriers

**DOI:** 10.1186/s12913-017-2464-0

**Published:** 2017-08-03

**Authors:** Quyen Le Thi Nguyen, Tuong Van Phan, Bach Xuan Tran, Long Hoang Nguyen, Chau Ngo, Huong Thi Thu Phan, Carl A. Latkin

**Affiliations:** 1 Institute for Global Health Innovation, Duy Tan University, Da Nang, Vietnam; 2 Institute of Health Management, Hanoi University of Public Health, Hanoi, Vietnam; 3 Institute for Preventive Medicine and Public Health, Hanoi Medical University, Hanoi, Vietnam; 4 Johns Hopkins Bloomberg School of Public Health, Baltimore, MD USA; 5 Authority of HIV/AIDS Control, Ministry of Health, Hanoi, Vietnam; 6 School of Medicine and Pharmacy, Vietnam National University, Hanoi, Vietnam

**Keywords:** Health insurance, HIV/AIDS, Coverage and barriers, Health finance, Policy

## Abstract

**Background:**

Health insurance (HI) plays an important role in ensuring the financial equity by the risk pooling mechanism and reducing the economic burden of healthcare for HIV/AIDS patients. However, there is a lack of evidence to clearly understand HI coverage in regard to people living with HIV (PLWH)**.** We conducted this study to explore the coverage and barriers of HI among PLWH in Vietnam.

**Methods:**

A cross- sectional study was conducted in multi-sites including 3 hospitals and 5 outpatient clinics in Hanoi and Nam Dinh in 2013. A convenience sampling approach was used to recruit the participants. A structured questionnaire was used to examine current status of using HI, lacking information about HI, feeling difficulties in accessing, using and paying HI. Multivariate logistic regression was conducted to examine factors associated with HI use and barriers.

**Results:**

Among 1133 HIV/AIDS patients, the coverage of HI was 46.0%. About 36.4% lacked information about HI, 21.0% felt difficulty in accessing HI. Meanwhile, the proportions of patients feeling difficulty in using HI and paying HI were 19.9 and 18.6%, respectively. Multivariate regression found that lacking information about HI and feeling difficulty in accessing HI were main barriers of having HI among PLWH.

**Conclusion:**

This study found a high proportion of PLWH was not covered by HI. Lacking information about HI and feeling difficulty in accessing HI were primary barriers that should be resolved via timely educational campaigns and consultations as well as supports from families in order to expand effectively the HI coverage.

## Background

Human Immunodeficiency Virus (HIV) is a chronic disease with multiple opportunity infections requiring lifetime treatment [1–5], which may increase risks of catastrophic health expenditures for households in the global context, especially in low and middle income countries as Vietnam [6–10]. A prior study reported that in Vietnam, nearly one-third of HIV-affected households experienced high health expenses despite the provision of free-of-charge antiretroviral therapy (ART) [11]. Additionally, as Vietnam is transitioning from a low-income to a lower middle-income country, international funds for HIV/AIDS programs are dramatically declining. This may lead to an issue that 94.1% of 67,057 HIV/AIDS patients receiving free ART under the funding will perhaps no longer be provided with free ART service [12]. Health insurance (HI) is considered a core solution for keeping HIV programs stable as well as having ART service remain accessible among people living with HIV/AIDS (PLWH).

HI plays an important role in reducing the economic burden of healthcare for patients and ensuring the financial equity by the risk pooling mechanism [13]. This mechanism refers to a financial protection against high health care cost through spreading risks amongst members of a pool [14]. Social HI (SHI) scheme covers most of HIV/AIDS services and treatments in high-income and even in middle-income countries with a higher rate of PLWH – such as Brazil, Mexico and Thailand [15]. In Taiwan, the highly active ART service has been free-of-charge for PLWH through the National HI program since 1997 [16]. In Thailand, SHI scheme has covered services and treatments for HIV-infected patients and voluntary counseling treatment for HIV high risk group to achieve equity in healthcare [17].

SHI was initially launched in Vietnam since 1992, and has been demonstrated to effectively reduce both direct and indirect medical cost of healthcare for household [18]. Given its enormous benefits, the Vietnam National Assembly has promulgated the Law on HI in 2008, and its amendments 2014, that mandated the enrollment of all citizens in SHI [19]. These laws introduce two co-existing national insurance schemes: a compulsory scheme and a voluntary scheme. The former includes employees of all type of firms (since 2009) and students (since 2010), while the latter includes farmers, self-employed, the elderly and children-over-six and others. SHI covers 100% health care cost for vulnerable populations such as the poor, the ethnic minorities and children under 6 years old; 95% for those who are close to the poverty line and retired people; and 80% for the others. HI Premium is estimated as 4.5% of base salary and equal to 653,400 VND (Vietnamese dollar, equivalent to 30 USD). People who close to poverty line and retired people have to pay 30% of HI Premium (190,000 VND or 9 USD) to get an HI card [20]. Additionally, people belonged to the poor and ethnic minorities, and children under 6 years old are subsidized by the state budget for free access to HI card. Noticeably, since 2013, with the aim of expanding HI coverage, voluntary HI is replaced by voluntary household-based HI that requires all family members having HI [21].

The Vietnamese government has set a goal of moving toward universal HI with the population coverage of 80% and reducing out-of-pocket health expenditure to under 40% by 2020 [22]. For PLWH, the Vietnamese government has commanded expanding HI to reach 100% insured PLWH. In order to encourage the enrollment of PLWH into SHI, HIV examination and treatment services such as ARVs and opportunistic infections drug, HIV and CD4 cell count test have been covered in Vietnamese HI scheme. Furthermore, many activities for consolidating and scaling up HI for PLHIV have being implemented in Vietnam. However, according to statistical reports of the Vietnam Authority of HIV/AIDS Control, the rates of insured PLWH varied from 30 to 50% across locations. Some barriers of accessing to HI among PLWH have been raised, namely: HI premium affordability and difficulties in participating HI scheme due to the lack of legal administrative procedures such as identity card and family record [23].

Working toward developing policy to expand HI in Vietnam requires systematic research for specific populations, particularly vulnerable groups such as PLWH. However, there has been scarcity of evidence about the coverage of HI among PLWH in Vietnam, the barriers to HI and the ways to resolve these problems. Therefore, we conducted this study to explore the coverage and barriers of HI among PLWH, and identify their associated factors in order to suggest contextualized solutions to promote the coverage of HI in Vietnam.

## Methods

### Study setting and participants

This study was conducted in eight outpatient clinics in Hanoi and Nam Dinh- two HIV/AIDS epicenters in Northern Vietnam with 18,108 and 21,500 PLWH, respectively.

The enrolled clinics comprised several levels of the health system including one national hospital (Bach Mai Hospital), one provincial hospitals (Nam Dinh provincial hospital), one provincial center (Nam Dinh provincial AIDS Center) and five district health centers (Hoang Mai, Long Bien, Dong Anh, Ha Dong, and Xuan Truong).

HIV patients who met following the inclusion criteria were invited to enroll into the study: 1) at least 18 years of age; 2) registered at selected outpatient clinics; and 3) able to complete the 30–40 min interview. For patients with HIV/AIDS who agreed to participate, informed consents were obtained and face-to-face interviews were conducted until our sample size was at least 200 patients per clinic at the national level and 100 patients per other sites. A total of 1133 adult patients receiving ART or pre-ART participated in this study. The participation rates were approximately 85–90% in all clinics.

### Measures and instruments

We utilized a structured questionnaire and recruited well-trained survey administrators to interview patients. The interviews were taken place in a designated counseling room and were conducted in Vietnamese language by Vietnamese interviewers. In this questionnaire, only an instrument entitled EUROQOL - 5 dimensions with five response levels (EQ-5D-5 L) was an international tool that was required a translation. We followed steps in a guideline of World Health Organization to translate from English to Vietnamese, which included forward translation, expert panel and back-translation [24].

**Fig. 1:**
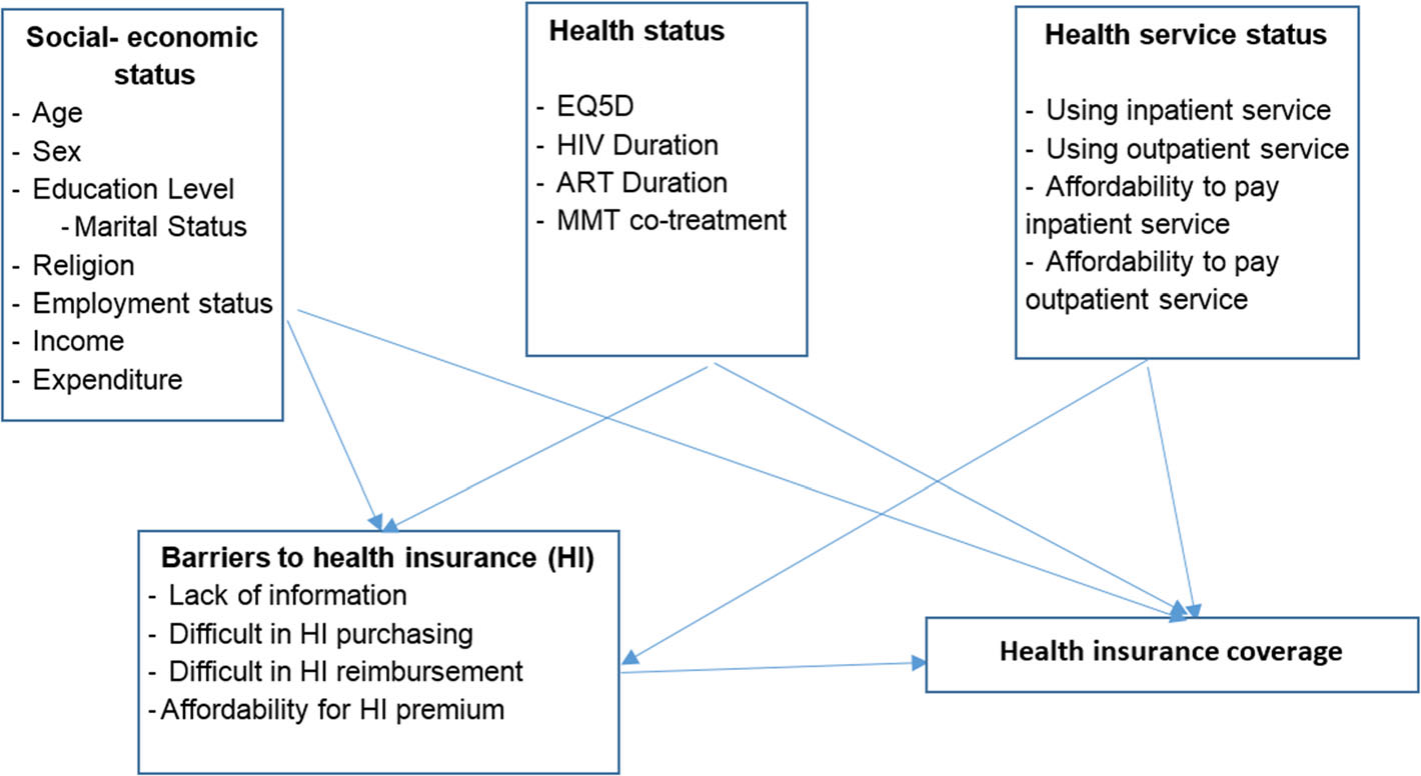
Conceptual framework about determinants of health insurance’s coverage and it-related barriers

The questionnaire included three main parts: socioeconomic characteristics (age, sex, education, marital status, religion, and employment status), health status and health services utilization, and current status of using HI and difficulties in accessing and using HI. These factors were selected based on reviewing prior literatures. The conceptual framework was displayed in Fig. 1. We first piloted the questionnaire with 20 PLWH to identify any text, content and logical issues. After piloting, because only minor text errors had been found that did not influence the responses, we decided to include people participating in pilot study into the whole study sample.

We examined health status of respondents by using EQ-5D-5 L including “no problem” “slightly”, “moderately”, “severely”, “extremely” [25]. Five dimensions included mobility, self-care, usual activities, pain/discomfort, and anxiety/depression. The Vietnamese version has been translated and validated in previous literatures [26–32].

Health services utilization was assessed by asking respondents if they received outpatient (excluding any HIV outpatient clinic visits for ARV medications) and inpatient care in the last 12 months. This approach has been applied in prior studies [27, 33–37]. Additionally, participants also were asked to report the location of the facilities where they received health services as well as the affordability of these services.

Respondents were then asked a question to assess their current HI status: “Do you have social HI?” After that, barriers to access and use HI were examined based on 4 dimensions: information, access to HI selling point, HI cost and paying health care services by HI. The questions used to measure this information were: “Do you lack information about HI?” “Did you feel any difficulties in accessing to HI selling points?” “Did you feel any difficulties in paying treatment cost by HI?” and “Did you feel any financial difficulties in paying HI cost?” Additionally, respondents were also asked a question about the expected time to pay HI.

### Data analysis

All data were entered into Microsoft Access and analyzed using Stata 12.0 software. We divided the participants into two groups (rural and urban), and examined the differences in characteristics between these group by using T-test and Chi-square test. To define catastrophic expenditures, we used a threshold of at least 40% total households’ monthly non-substances expenditure [38].

Finally, we used multivariable logistic models to explore the influence of socio-economic characteristic, health status and health services utilization on the HI status as well as barriers to access health. We used a stepwise backward selection strategy to develop a reduced model from the original full model. Log-likelihood ratio test at a *p*-value of 0.2 was applied to remove non-significant factors [17]. A *p*-value <0.05 was set as level of statistical significance.

## Results

Of the 1133 participants with an average age of 35.5 years old (SD = 6.9), 58.7% were male. The majority of patients lived with spouse/partner (61.2%), had cult of ancestors (88.4%). There were 42.7% of respondents completing high school or above. One-fifth of the sample were unemployed. The majority of respondents (96.0%) were currently on ART treatment (Table 1).

**Table 1:** Demographic characteristics of respondents

	Rural		Urban		Total		*P*-value
	Number	Percent	Number	Percent	Number	Percent	
Sex							
Male	145	56.2	520	59.4	665	58.7	0.36
Female	113	43.8	355	40.6	468	41.3	
Education							
< Elementary	53	20.5	179	20.5	232	20.5	0.03
Secondary	109	42.3	309	35.3	418	36.9	
High	80	31	282	32.2	362	32	
> Vocational	16	6.2	105	12	121	10.7	
Marital status							
Single	26	10.1	143	16.3	169	14.9	0.01
Live with spouse/partner	178	69	515	58.9	693	61.2	
Divorced/widow	54	20.9	217	24.8	271	23.9	
Religion							
Cult of ancestors	222	86.1	779	89.03	1001	88.4	0.03
Buddhism and Protestant	12	4.6	52	5.94	64	5.6	
Catholic	24	9.3	44	5.03	68	6.0	
Employment							
Unemployed	47	18.2	185	21.1	232	20.5	<0.01
Self-employed	98	38	371	42.4	469	41.4	
White collars	13	5	67	7.7	80	7.1	
Workers, Farmers	94	36.4	188	21.5	282	24.9	
Other jobs	6	2.3	64	7.3	70	6.2	
ART							
Currently receiving ART	249	98.0	801	95.4	1050	96.0	0.06
Pre-ART	5	2.0	39	4.6	44	4.0	
	Mean	SD	Mean	SD	Mean	SD	
Age	35.6	6.6	35.5	7	35.5	6.9	0.79

A relatively high proportion of participants experienced anxiety/depression (44.9%), pain/discomfort (37.7%). Of the sample, 16.6% reported problems in usual activities, 20.5% experienced problems in mobility and a low rate of respondents having problems with self-care (9.7%). People living in urban area had higher rates of problems in five dimensions than that among rural people. Regarding inpatient services utilization, 18.4% reported using these services, 55.5% admitted to the central health clinics and more than half of the sample could pay full charges. Inpatient service usage in city dwellers was two times higher than that of rural ones. As for outpatient services, 29.8% reported using these services, 29.0% admitted to the central hospital and more than two-third of patients were fully affordable to this health care service (Table 2).

**Table 2:** Behaviors, health status and health service utilization of respondents

	Rural		Urban		Total		*P*-value
	Number	Percent	Number	Percent	Number	Percent	
EQ5D5L							
Having problem in mobility	33	12.8	199	22.7	232	20.5	<0.01
Having problem in self-care	12	4.7	98	11.2	110	9.7	<0.01
Having problem in usual activities	26	10.1	162	18.5	188	16.6	<0.01
Pain/Discomfort	85	33.0	342	39.1	427	37.7	0.07
Anxiety/Depression	104	40.3	405	46.3	509	44.9	0.09
Inpatient service use	28	10.9	180	20.6	208	18.4	<0.01
Facility for inpatient use							
Central	10	38.5	101	58.1	111	55.5	0.10
Province	9	34.6	49	28.2	58	29.0	
Others	7	26.9	24	13.8	31	15.5	
Affordability to pay inpatient services							
Full payment	15	57.7	87	51.2	102	52.0	0.72
Unaffordable	8	30.8	51	30.0	59	30.1	
Partly payment	3	11.5	32	18.8	35	17.9	
Outpatient service use	76	29.5	262	29.9	338	29.8	0.88
Facility for outpatient use							
Central	15	20.6	82	31.4	97	29.0	0.01
Province	17	23.3	64	24.5	81	24.3	
District	25	34.3	43	16.5	68	20.4	
Private	9	12.3	52	19.9	61	18.3	
Others	7	9.6	20	7.7	27	8.1	
Affordability to pay outpatient services							
Full payment	49	84.5	172	75.1	221	77.0	0.26
Unaffordable	4	6.9	33	14.4	37	12.9	
Partly payment	5	8.6	24	10.5	29	10.1	

Table 3 presents the current coverage and barriers to access to social HI among respondents. About 46.0% of respondents had HI and this rate in the rural was higher than that among urban people. Barriers of accessing to HI included: lack of information about HI (36.4%), difficulty of access HI (21.0%), difficulty of HI use (19.9%), and difficulty to make HI payment (18.6%). The percentage of urban citizens reported having HI barriers was higher than their rural counterparts in all categories, except for the “difficult to pay HI” category. A quarter of participants preferred to pay HI in multiple installments per year.

**Table 3:** Current coverage and barriers to social HI among respondents

	Rural		Urban		Total		*P*-value
	Number	Percent	Number	Percent	Number	Percent	
Having HI	136	52.7	385	44	521	46.0	0.01
Lack of Information about HI							
Yes	65	25.8	335	39.5	400	36.4	<0.01
No	187	74.2	513	60.5	700	63.6	
Feel difficulty in accessing HI							
Yes	44	17.7	186	22.0	230	21.0	0.15
No	204	82.3	660	78.0	864	79.0	
Feel difficulty in using HI							
Yes	46	18.6	170	20.3	216	19.9	<0.01
No	177	71.4	392	46.9	569	52.5	
Unknown	25	10.1	274	32.8	299	27.6	
Feel difficulty in paying HI							
Yes	47	19.2	154	18.5	201	18.6	<0.01
No	169	69.0	467	56.1	636	59	
Unknown	29	11.8	212	25.5	241	22.4	
Expected time to pay HI							
Monthly	14	5.6	89	10.5	103	9.4	0.149
Quarterly	21	8.4	71	8.4	92	8.4	
Every 6 months	22	8.8	60	7.1	82	7.5	
Annually	160	64	502	59.3	662	60.4	
Unknown	33	13.2	125	14.8	158	14.4	

Table 4 presents factors associated with owning HI. People who had insufficient information about HI (OR = 0.41; 95% CI = 0.24–0.69) or difficulty to access HI (OR = 0.47; 95% CI = 0.23–0.95) were less likely to have HI. Meanwhile, having university degrees or higher (OR = 15.83; 95% CI = 2.87–87.15) and living with spouses (OR = 2.71; 95% CI = 1.59–4.63) increased significantly the likelihood of having HI among patients. In addition, the likelihood of having HI among respondents suffering catastrophic expenditure were 1.79 times higher than other people (OR = 1.79; 95% CI = 1.05–3.05).

**Table 4:** Factors associated with the use and barriers of SHI among respondents

	Having HI		Lack of information about HI		Feel difficulty in accessing HI		Feel difficulty in using HI		Feel difficulty in paying HI	
	AOR	95%CI	AOR	95%CI	AOR	95%CI	AOR	95%CI	AOR	95%CI
Gender (Female vs. Male)							4.97**	1.15; 21.57		
Education (vs. Illiterate)										
• High school							0.49**	0.24; 1.00		
• Vocational			0.10**	0.01; 0.81						
• ≥ University	15.83***	2.87; 87.15					5.62***	1.58; 19.94		
Marital status (vs. Single)										
• Living with spouse/partner	2.71***	1.59; 4.63	0.67*	0.43; 1.04	0.53**	0.32; 0.88				
• Divorces							4.77***	1.98; 11.47		
• Widow	6.36*	0.77; 52.82								
Religion (vs. No)										
• Catholic	2.81**	1.13; 6.98								
Having problem in self-care (Yes vs. No)									2.26**	1.01; 5.05
Pain/Discomfort (Yes vs. No)			2.74***	1.73; 4.32	1.58*	0.95; 2.62				
Catastrophic health expenditure (Yes vs. No)	1.79**	1.05; 3.05					1.81*	0.94; 3.48		
Lack of information about HI (Yes vs. No)	0.41***	0.24; 0.69								
Feel difficulty in accessing HI (Yes vs. No)	0.47**	0.23; 0.95								

*** *p* < 0.01, ** *p* < 0.05, * *p* < 0.1

Table 4 also indicates that having vocational training degree were negatively associated with lacking information about HI (OR = 0.10; 95% CI = 0.01–0.81), while experiencing pain/discomfort were positively related to having inadequate information (OR = 2.74; 95% CI = 1.73–4.32). Regarding feeling difficulty to access HI, people living with spouse/partners were approximately two times less than single patients in confronting difficult to access HI (OR = 0.53; 95% CI =0.32–0.88). For difficult to use HI, people who were female, having university degree or higher, living with spouse/partner and experiencing catastrophic health expenditure had higher likelihood to face difficulties in using HI. Finally, only having problems in self-care was found to be positively associated with having difficulties in paying HI (OR = 2.26; 95% CI = 1.01–5.05).

## Discussion

This study examined HI coverage and barriers among PLWH, which contribute to the existing evidence for planning strategies to increase the coverage of HI in Vietnam. Our results show that the rate of PLWH having HI was low while the rates of PLWH who could not afford health care services remained relatively high. In addition, this study also depicts high proportions of PLWH experiencing a lack of information about HI and facing problems with accessibility, affordability and usage of HI. Findings from multivariate analysis suggest that several potential implications to promote the coverage of HI among PLWH in Vietnam.

PLWH and their families are at high risk of financial burden due to out-of-pocket payments for health care even when ART is provided freely in Vietnam. Prior studies indicated that more than one third of households having PLWH suffered catastrophic expenditures [11, 39, 40]. In our study, nearly half and more than one fifth of respondents were unable to fully pay inpatient and outpatient services, respectively. This problem might be more substantial as in the coming years, ART will no longer be free (101 USD for the 1st line and 1049 USD for 2nd line of ARV regimen per year) due to the rapid decline of international aids [12].

Given benefits as covering HIV-related services, having HI is necessary for protecting PLWH from any financial consequences of healthcare payments. However, HI covered only approximately half of our respondents. This coverage rate was much lower than that among PLWH in other countries such as America (70.4–73.5%) [41] and Thailand (72%) [42]. The rate of insured PLWH in our study was also much lower than that in the general Vietnamese population (66.8%) (2012) [19]. This figure implies that despite strong commitments of the Vietnam Government to cover all HIV-related services in HI schemes, it is still a long road to reach universal coverage among PLWH in Vietnam [16].

Notably, one significant problem is that patients might prefer to buy HI only when they suffer sickness [21, 43]. There are several reasons behind this decision such as long waiting time, complicated administrative procedure, low service quality, and high informal payments for medical staffs [21, 44, 45]. In current study, we found that people experiencing catastrophic expenditures due to healthcare in the last 12 months were more likely to have HI at the time of interview. We assumed that the patients might be aware of the advantages of HI in preventing high out-of-pocket payments and decide to buy HI afterward. This is still a substantial problem when limiting the risk pooling role of HI; otherwise, HI could not be used as a preventive mechanism for financial burden.

Addressing issues as regards lacking information about HI could potentially encourage the enrollment of PLWH in HI schemes, which is demonstrated in previous studies [46, 47]. Multivariate analysis indicates that people having insufficient information were less likely to have HI. In our sample, more than one-third of respondents reported a lack of information about HI. Having inadequate information in our study means not only having insufficient knowledge about HI, but also perceiving limitations of HI via unofficial sources as well as negative attitudes from HI users (e.g. from friends or relatives who already used HI for health service utilization). This information might mislead PLWH about the benefits of HI, resulting in the feeling of difficulties in accessing, using and paying HI, and eventually, preventing the intention to pay and use HI of PLWH, particularly among those not yet owning a HI card. Moreover, respondents shared that they worried about their confidentiality when having HI and they were not aware of their rights in protecting their privacy. Literature documented that HIV/AIDS stigma was a barrier in using HI among PLWH because their HIV status was disclosed due to the requirement of the test form or prescription [48–50]. However, it should be noted that the information of PLWH is protected by many legal regulations (e.g. Law on HIV/AIDS prevention and prevention and Law on Medical examination and treatment) [51, 52]. Therefore, informing accurate knowledge about the benefits of HI in regard to reimbursement, quality of service and rights of information protection is very necessary to motivate PLWH participating in HI.

Along with lacking information, we recognized that feeling difficulty in accessing HI was also a main challenge to expand the coverage of HI in PLWH population. Noticeably, we found that people living with spouse/partner did not face this barrier compared to single ones. As mentioned before, stigma is still an obstacle in having HI, including meeting with health staffs or people who are responsible for HI in the community [48–50]. As such, spouses/partners of patients can help them to access and buy HI. This is the principle of household HI that one person can purchase HI for his/her family members, which is expected to be implemented in the future. However, due to the replacement of individual HI by household HI that required the participation of all family member, HI premium is reported as a financial burden for patients and their family [23]. In fact, about 19% patients reported feeling difficulty in paying HI. Therefore, to reduce the economic burden, it is recommended that HI premium should be paid in multiple installments per year and a quarter of the sample preferred to pay HI monthly, quarterly or bi-annually. In addition, financial support policies for the poor, people who close to poverty line and other vulnerable people among PLWH should be continued and promoted simultaneously.

In this study, patients having university degree or higher were more likely to be insured. We assumed that people with higher education might highly perceive the importance of HI and pay HI for financial protection [44, 53, 54]. Moreover, they have a higher likelihood to have stable jobs, and their HI are paid mandatorily by their employers according to the Vietnam Labor Code [55]. In addition, they were also likely to feel difficulty in using HI in health facilities, while people having lower education felt less difficulty in utilizing HI. It is due to the fact that people attaining high education had higher expectation for the healthcare services; however, when using these services, they might confront some aforementioned limitations of HI-related service and then feel difficulties and unsatisfied. This issue is well-documented in various populations [56–58]. Similar reasons could be used to explain the figures that female and divorced patients were more likely to feel difficulty in using HI compared to male and single ones, respectively [57, 59, 60].

Several implications should be considered from the study. First, campaigns to enhance communication and providing information about HI for PLWH are recommended. Integration of counseling as well as delivering leaflet, guidebook and pictures about HI when patients visit HIV outpatient clinics should also be considered. In addition, information about HI should be broadened for the people having high at risk of HIV at methadone maintenance treatment clinics, HIV counselling and testing sites, as well as through peer outreach channels. Second, to effectively expand HI for PLWH, their families should be involved to support them in accessing and paying HI via household HI. In addition, policy makers (HI agencies) could allow users to pay HI premium in multiple installments per year to increase the affordability of HI premium. In health facilities, regulations about using discreet code instead of disease names and simplifying the administrative procedures should be developed to reduce the barriers in using HI among PLWH.

Our study has several strengths. First, the study recruited a relatively large number of respondents from multiple sites across levels of health system and geographical areas of Vietnam. Second, the study addressed the pressing issue of HIV/AIDS programs in Vietnam. However, our study also has several limitations. First, this study was designed as a cross-sectional study, thus we could not determine how the barriers in HI reimbursement, HI premium, HI purchasing and lack of HI information influence patients’ insurance coverage over time. Second, our study is limited in exploring the details of these barriers as well as the need and the demand of these patients that can better support for the HI policy developments, and can be included in further study of a quantitative combining with qualitative. Third, we collected self-reported information, which might lead to recall bias. Finally, we utilized convenience sampling technique, that may limit the generalization of this study to other settings.

## Conclusion

To conclude, this study found a high proportion of PLWH were not covered by HI. Lacking information about HI and feeling difficulty in accessing HI were primary barriers that should be resolved via timely educational campaigns and consultations as well as supports from families in order to expand effectively the HI coverage.

## Abbreviations

AIDS: Acquired immune deficiency syndrome
ART: Antiretroviral therapy
HI: Health insurance
HIV: Human immunodeficiency virus
PLWH: People living with HIV
SHI: Social health insurance
